# Elevated Humidity Impairs Evaporative Heat Loss and Self‐Paced Exercise Performance in the Heat

**DOI:** 10.1111/sms.70041

**Published:** 2025-03-19

**Authors:** Felicity M. Bright, Brad Clark, Ollie Jay, Julien D. Périard

**Affiliations:** ^1^ Research Institute for Sport and Exercise University of Canberra Canberra Australia; ^2^ Heat and Health Research Centre, Faculty of Medicine and Health The University of Sydney Sydney Australia

**Keywords:** absolute humidity, cardiovascular strain, hyperthermia, relative humidity, thermal strain, time trial

## Abstract

This study investigated the effects of absolute humidity on heat dissipation and subsequent thermal, cardiovascular, and performance responses during self‐paced exercise in the heat. Twelve trained male cyclists performed a 700‐kJ time trial in four different humidity conditions (Low: 1.6 kPa, Moderate: 2.5 kPa, High: 3.5 kPa, and Very high: 4.5 kPa) in 33°C. The gradient in partial water vapor pressure at the saturated skin surface and in air, which determines sweat evaporation, decreased significantly with increasing humidity (Low: 3.53 ± 0.30 kPa, Moderate: 2.74 ± 0.24 kPa, High: 1.99 ± 0.20 kPa, Very high: 1.19 ± 0.16 kPa; *p* < 0.001). The maximum evaporative capacity of the environment (*E*_max_) also decreased with greater humidity (Low: 309 ± 26 W m^−2^, Moderate: 240 ± 21 W m^−2^, High: 175 ± 18 W m^−2^, Very high: 104 ± 14 W m^−2^; *p* < 0.001), as did sweating efficiency (*S*_eff_) (Low: 0.50 ± 0.13, Moderate: 0.39 ± 0.10, High: 0.28 ± 0.09, Very high: 0.16 ± 0.04; *p* ≤ 0.003). Power output was similar between Low (260 ± 33 W) and Moderate humidity (257 ± 27 W; *p* = 0.999), but lower in Very high (222 ± 37 W) than in all other conditions (*p* < 0.001) and lower in High (246 ± 31 W) than in the Low and Moderate humidity (*p* < 0.001). Peak core temperature was higher in Very high (39.49°C ± 0.56°C) than in Low (38.97°C ± 0.44°C; *p* < 0.001), Moderate (39.04°C ± 0.39°C; *p* = 0.002) and High humidity (39.12°C ± 0.47°C; *p* = 0.010). Mean skin temperature was higher with elevated humidity (*p* < 0.001) and mean heart rate was not significantly different between conditions (*p ≥* 0.056). These data indicate that reductions in evaporative potential and efficiency with elevated humidity exacerbate thermal and cardiovascular strain during self‐paced cycling in the heat, resulting in marked performance impairments.

## 1 Introduction

Humidity influences the potential for sweat to evaporate from the skin surface, which is the primary avenue for heat loss when ambient temperature approaches or exceeds skin temperature [1]. Humidity is often reported as relative humidity (RH), which is the amount of water vapor in the air expressed as a percentage of the amount of water vapor needed to achieve saturation at a given temperature. However, it is absolute humidity, the total amount of water vapor in the air, that determines the potential for evaporative heat loss [2]. Accordingly, the evaporation of sweat is determined by the difference in the partial pressure of water vapor at the saturated skin surface and in the air (i.e., *P*_sk,sat_–*P*_a_ gradient [2, 3]). In dry conditions (*P*_a_ of ≤ 1.5 kPa), a wide *P*_sk,sat_–*P*_a_ gradient increases the maximum evaporative capacity of the environment (*E*_max_) and sweat is more easily evaporated from the skin surface. Conversely, in humid conditions (e.g., *P*_a_ of 4 kPa [4]), a narrow *P*_sk,sat_–*P*_a_ gradient impedes the capacity for evaporative heat loss with large volumes of sweat coalescing and dripping off the skin, and not contributing to body heat loss [2, 5]. Hence, in hot and humid conditions, impairments in exercise capacity (i.e., time to exhaustion) relate to an inability to dissipate heat and the precipitated rise in thermal, cardiovascular, and perceptual strain (i.e., rating of perceived exertion [RPE] and thermal discomfort) [6, 7]. For example, when cycling at 70% of peak oxygen uptake (V̇O_2peak_) in 30°C, volitional exhaustion occurred 14 and 22 min earlier at absolute humidities of 2.55 and 3.39 kPa (60% and 80% RH) respectively, compared with 1.02 kPa (24% RH) [6]. In the 2.55 and 3.39 kPa conditions, the reduction in exercise capacity was associated with a greater rate of rise in rectal temperature (*T*_re_) and a higher mean skin temperature (*T*_sk_).

During self‐paced exercise (i.e., 30–60 min time trial), it was recently demonstrated that performance at a similar absolute humidity of 1.96 kPa did not significantly differ when conducted in ambient temperatures of 18°C and 27°C (95% and 55% RH, respectively), but was impaired at an ambient temperature of 36°C (33% RH) [8]. Performance was further impaired in 36°C at an absolute humidity of 3.92 kPa (66% RH). The impairment in performance during self‐paced exercise under heat stress is characterized by a gradual decline in work rate in response to a greater rise in thermal (i.e., *T*_re_ and *T*_sk_) and cardiovascular (i.e., elevated heart rate) strain than during exercise in cooler conditions [9, 10]. The elevated cardiovascular response leads to a gradual reduction in V̇O_2peak_ and an increase in relative exercise intensity (i.e., %V̇O_2peak_) for work performed at a given absolute intensity (i.e., work rate [11, 12]). As such, work rate must be reduced to maintain relative exercise intensity within a narrow range, or optimal performance intensity [12]. However, the extent to which different humidity levels impact self‐paced exercise performance and pacing in hot conditions remains to be elucidated. Given that endurance sports are undertaken in hot and humid environments (e.g., 2019 Doha World Athletics Championships women's marathon: 32°C, 3.52 kPa [74% RH] [13], 2020 Tokyo Olympics men's cycling road race: 32°C, 3.19 kPa [67% RH] [14]), a greater understanding and ability to estimate the impact of different humidity levels on physiological and perceptual responses, along with their performance implications, would benefit athlete training and competition preparation.

Therefore, this study investigated the influence of four different absolute humidities (Low: 1.6 kPa, Moderate: 2.5 kPa, High: 3.5 kPa, and Very high: 4.5 kPa) on 700‐kJ cycling time trial performance at an ambient temperature of 33°C. To identify the pathway(s) via which exercise performance is impacted by humidity, adjustments in thermal, cardiovascular, and perceptual responses were investigated, along with heat balance using partitional calorimetry. It was hypothesized that a reduction in evaporative potential associated with elevated water vapor in the air and the consequent narrowing of the *P*_sk,sat_–*P*_a_ gradient would exacerbate physiological and perceptual strain, resulting in progressively larger decrements in performance (i.e., power output) during the time trials with elevated humidity.

## 2 Methods

### 2.1 Participants

Twelve non‐heat acclimatized male cyclists volunteered for this study (mean ± SD: age 41 ± 9 years; height 1.79 ± 0.05 m; body mass 76.3 ± 9.3 kg; V̇O_2peak_ 4.7 ± 0.1 L min^−1^). Participants were trained (Performance Level 3 [15]) and free of any injury or illness. All participants gave written and informed consent and completed an Adult Pre‐exercise Screening Tool [16] before study commencement. The study was approved by the University of Canberra Human Research Ethics Committee (Project ID: 2116), and all procedures conformed to the standards of the Declaration of Helsinki.

### 2.2 Experimental Design

The study followed a counterbalanced cross‐over design, with each participant completing two pre‐experimental trials and four experimental trials in the environmental chamber (QRA International Pte Ltd., Singapore) at the University of Canberra Research Institute for Sport and Exercise between the months of April and November. All trials were separated by a minimum of 48 h, each performed at the same time of day. In pre‐experimental trials, participants completed an incremental exercise test to volitional exhaustion in temperate conditions (~20°C and ~40% RH) for the determination of V̇O_2peak_. Following a 30‐min period of passive rest, participants completed a 350‐kJ time trial, during which they were familiarized with the experimental procedures and measurements. This procedure was repeated to determine the coefficient of variation for time trial performance. After completing the pre‐experimental trials, participants completed four experimental trials consisting of a 700‐kJ cycling time trial. The environmental characteristics of the four experimental trials are presented in Table 1. During all trials, a frontal‐facing air flow of 3.4 ± 0.27 m s^−1^ was provided by a pedestal fan (Dynabreeze IP65 Pedestal Fan 400 mm, Tradeware Australia, Regency Park, Australia) placed 1.5 m from the cycle ergometer.

**TABLE 1:** Environmental characteristics (absolute humidity, ambient temperature, and relative humidity) for the low, moderate, high, and very high humidity conditions.

Humidity condition	Absolute humidity (kPa)	Ambient temperature (°C)	Relative humidity (%)
Low	1.6 ± 0.2	32.5 ± 0.8	32.9 ± 4.2
Moderate	2.5 ± 0.2	32.8 ± 0.9	50.5 ± 1.9
High	3.5 ± 0.2	32.8 ± 0.9	69.9 ± 2.7
Very high	4.5 ± 0.1	33.1 ± 0.8	88.4 ± 3.2

All trials were completed on a SmartIT Trainer cycle ergometer (SRM GmbH, Jülich, Germany). Participants adjusted the saddle and handlebars to their preferred position during the first trial, which remained constant for the other trials. Throughout the time trials, participants were aware of work completed, with verbal encouragement provided during the final 10 kJ. In each trial, participants wore cycling shorts, socks and cycling shoes. Environmental characteristics throughout each trial were measured using an environmental monitor (5400 Weather Meter; Kestrel Meters, Boothwyn, PA) placed directly in front of the cycle ergometer at a height of 1.2 m. Before visiting the laboratory, participants were required to refrain from strenuous exercise, and caffeine and alcohol consumption for at least 24 h. They were also provided with and asked to complete a 24‐h food diary prior to the first experimental trial and to replicate their diet before the remaining trials.

### 2.3 Incremental Exercise Tests

The incremental exercise test consisted of three 5‐min stages of submaximal cycling at 120, 160, and 200 W, after which power output increased by 25 W min^−1^ until volitional exhaustion. Expired gases were collected via a one‐way valve (Hans Rudolph Inc., Shawnee, KS, USA) and analyzed using a stationary metabolic gas analyzer (TrueOne, Parvomedics, Sandy, UT, USA). Heart rate and RPE were recorded at the end of each 5‐min submaximal stage, with heart rate and RPE recorded at 25 and 50 W increments thereafter, respectively.

### 2.4 Experimental Protocol

Upon arrival to the laboratory (~60 min prior to time trial commencement), the weight of participants' cycling shorts and socks were measured, after which they provided a urine sample for the assessment of urine specific gravity (USG: PEN‐Urine S.G., ATAGO, Tokyo, Japan). If USG was > 1.020, participants were instructed to consume 5 mL kg^−1^ of body mass of water in the 40–60 min preceding the time trial. Participants then self‐inserted a general‐purpose thermistor probe (TM400, Covidien, Mansfield, MA, USA) 10 cm beyond the anal sphincter for the measurement of *T*_re_. Once participants were changed into their cycling shorts, body mass was recorded using a platform scale (KW‐4050‐150+; KW Industrial Platform Scale, VIC, Australia). Participants were then equipped with a heart rate monitor chest strap (T‐31 Polar Electro, Lake Success, NY, USA) and skin temperature sensors (iButtons, Maximum Integrated Products, San Jose, CA, USA). After 5 min of supine rest in cool conditions (18°C–20°C), baseline measures of heart rate, *T*_re_, *T*_sk_, and thermal comfort were recorded. Participants then moved to the environmental chamber to rest for a further 10 min (Table 1). Next, participants completed a 10‐min standardized warm‐up consisting of cycling for 4 min at 40% of the peak power output determined during the incremental test, 3 min at 50%, 2 min at 60% and 1 min increasing from 60%–80%. A further 3 min of seated rest on the ergometer or unloaded spinning completed the warm‐up. Participants were then instructed to complete the 700‐kJ time trial as quickly as possible. Power output and cadence were continuously recorded on an SRM Powercontrol 8 (SRM GmbH, Jülich, Germany).

### 2.5 Thermal and Cardiovascular Measurements

Throughout the baseline rest period, warm‐up, and during the time trial at every 10% of work completed, T_re_ was recorded (Squirrel SQ2010, Grant Instruments, Cambridge, England). Weighted mean T_sk_ was calculated as chest (30%), shoulder (30%), thigh (20%) and calf (20%) [17]. The core‐to‐skin temperature (*T*_re_‐to‐*T*_sk_) gradient was calculated by subtracting *T*_sk_ from *T*_re_ at rest and at every 10% of work completed. Heart rate was measured continuously with a Polar Chest Strap (T‐31 Polar Electro, Lake Success, NY, USA), and participants were fitted with a mouthpiece and nose clip to collect and analyze expired gases at 10%, 40%, 70%, and 90% of work completed. Expired gases were collected for 90 s, averaged across the four measurement time points, and used to calculate metabolic heat production (*H*_prod_).

### 2.6 Heat Balance Estimations

Equations used to calculate the *P*_sk,sat_–*P*_a_ gradient, *E*_max_, rate of evaporation required for heat balance (*E*_req_), rate of convective and radiative heat exchange (*C* + *R*), rate of respiratory convective and evaporative heat loss (*C*_res_ + *E*_res_), *H*_prod_, and sweating evaporative efficiency (*S*_eff_) are presented in the Appendix A.

### 2.7 Perceptual and Hydration Measurements

Participant RPE [18] and thermal comfort [19] were measured at rest (except for RPE) and at 10%, 30%, 50%, 70%, 90%, and 100% of work completed. During the time trials, participants consumed room‐temperature water ad libitum. Whole‐body sweat rate was calculated by subtracting post‐trial body mass from pre‐trial body mass, corrected for fluid ingested and sweat trapped in clothing.

### 2.8 Statistical Analysis

An a priori power analysis for sample size was conducted for differences in time to exhaustion during constant work rate exercise studies between the extreme conditions of hot‐dry and hot‐humid environments using a Cohen's *d* of 1.32 [6] and 1.63 [7]. With an alpha of 0.05 and power of 0.80, the estimated sample size needed for repeated measures of within‐group comparisons was five (G Power software version 3.1.9.4). Given the additional RH conditions and self‐paced nature of the intervention, we recruited 12 participants. All statistical analyses were performed using SPSS Software (IMB SPSS Statistic Version 25). Linear mixed models were fitted with all thermal, physiological, perceptual, and performance variables as dependent variables. A linear mixed model was also performed to test for a trial order effect. Condition and work completed (i.e., time) effects were included in the models as fixed, and participant as a random effect for the dependent variables of power output, *T*_re_, *T*_sk_, *T*_re_‐to‐*T*_sk_ gradient, heart rate, V̇O_2_, RPE, and thermal comfort. Only condition effects were included in the models as fixed, and participant as a random effect for baseline *T*_re_, *T*_sk_, USG, and heart rate, as well as body mass loss and *P*_sk,sat_–*P*_a_ gradient, *H*_prod_, *E*_max_, *E*_req_, *C* + *R*, *C*_res_ + *E*_res_, and *S*_eff_. Where significant effects were established, pairwise comparisons were conducted using *post hoc* analysis with Bonferroni adjustment for multiple comparisons. Values of *p <* 0.05 were deemed statistically significant, and effect sizes were interpreted as small (0.2), medium (0.5) and large (0.8) [20]. All values are expressed as mean ± standard deviation (SD).

## 3 Results

### 3.1 Pre‐Experimental V̇O_2peak_ Tests and Familiarization Time Trial

V̇O_2peak_ in pre‐experimental test one (4.63 ± 0.38 L min^−1^) and test two (4.74 ± 0.32 L min^−1^) was not significantly different (*p* = 0.055). The respiratory exchange ratio was also similar between tests (1.06 ± 0.06 and 1.07 ± 0.08; *p* = 0.539), as was the maximum heart rate (173 ± 14 beats min^−1^ and 174 ± 14 beats min^−1^; *p* = 0.353). RPE values upon reaching volitional exhaustion were 18.9 ± 1.8 and 19.0 ± 1.7; *p* = 0.745. There was no significant difference in mean power output between the two familiarization time trials (267 ± 29 W and 271 ± 30 W; *p* = 0.174) with a coefficient of variation of 3.3%.

### 3.2 Time Trial Performance

**FIGURE 1:**
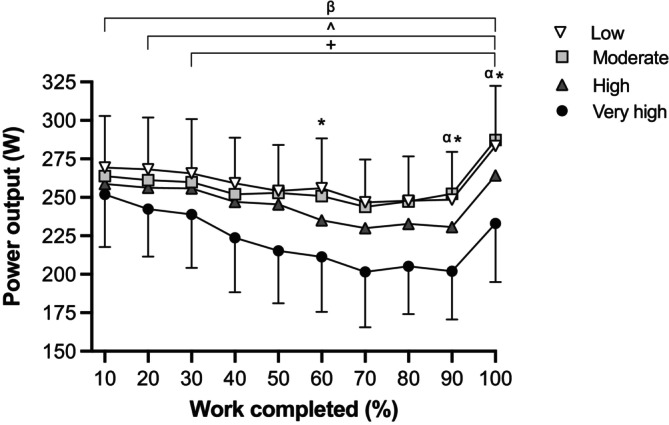
Power output during a 700‐kJ time trial at 33°C with Low, Moderate, High, and Very high absolute humidity. ^β^Significant difference between Low and Very high (*p* ≤ 0.037). ^^^Significant difference between Moderate and Very high (*p* ≤ 0.019). ^+^Significant difference between High and Very high (*p* ≤ 0.047). *Significant difference between Low and High (*p* ≤ 0.033). ^α^Significant difference between Moderate and High (*p* ≤ 0.005). All values are expressed as means and SD. Differences were observed using a linear mixed model.

Counterbalancing between conditions was achieved as there was no trial order effect for time trial duration (*p* = 0.657). A significant condition effect (*p* < 0.001) was identified for trial duration, with a slower trial in Very high (52:54 ± 7:14 min) than Low (46:16 ± 4:20 min; *p* < 0.001), Moderate (45:54 ± 3:01 min; *p* < 0.001) and High humidity (48:17 ± 4:54 min; all *p* = 0.001; *d* = 0.75 to 1.26). Significant condition (*p* < 0.001), time (*p* < 0.001) and interaction (*p* = 0.004) effects were identified for power output during the time trials (Figure 1).

Throughout the time trial, power output was similar between Low and Moderate humidity (*p* = 0.999; *d* = −0.14 to 0.26), and for the first 50% of the time trial, power output was similar between Low and High humidity (*p* > 0.05; *d* = 0.30 to 0.42). Only at 60%, 90%, and 100% of the time trial was power output lower in High than Low humidity (*p* ≤ 0.033; *d* = −0.71 to −0.46). From 90% of the time trial, power output was lower in High than Moderate humidity (*p* ≤ 0.005; *d* = −0.74 to −0.61). From 30% of work completed, power output was lower in Very high than High humidity (*p* ≤ 0.047; *d* = −0.96 to −0.56). From 20% of work completed, power output was lower in Very high than Moderate humidity (*p* ≤ 0.019; *d* = −1.81 to −0.67) and from the commencement of exercise (10% of work completed), power output was lower in Very high than Low humidity (*p* ≤ 0.037; *d* = −1.49 to −0.52).

Significant condition (*p* < 0.001) and time (*p* < 0.001) effects were identified for cadence, but there was no interaction effect (*p* = 0.988). Cadence was significantly higher in Low (95 ± 7 rev min^−1^) than High (92 ± 9 rev min^−1^; *p* < 0.001; *d* = 0.28) and Very high humidity (90 ± 87 rev min^−1^; *p* < 0.001; *d* = 0.60), but not Moderate (93 ± 9 rev min^−1^; *p* = 0.076; *d* = 0.15), which was also not significantly higher than High humidity (*p* = 0.172; *d* = 0.12). Cadence was higher in both Moderate (*p* < 0.001; *d* = 0.40) and High humidity (*p* < 0.001; *d* = 027), compared to Very high.

### 3.3 Heat Balance

A significant condition effect was identified for *P*_sk,sat_–*P*_a_ gradient (*p* < 0.001), *E*_max_ (*p* < 0.001), *E*_req_ (*p* < 0.001), *C* + *R* (*p* < 0.001), *C*_res_ + *E*_res_ (*p* < 0.001), *H*_prod_ (*p* < 0.001) and S_eff_ (*p* < 0.001; Table 2). A narrower *P*_sk,sat_–*P*_a_ gradient was identified in Very high than Low (*p* < 0.001; *d* = −9.69), Moderate (*p* < 0.001; *d* = −7.61) and High humidity (*p* < 0.001; *d* = −4.43), in High than Low (*p* < 0.001; *d* = −5.96) and Moderate humidity (*p* < 0.001; *d* = −3.36), and in Moderate than Low humidity (*p* < 0.001; *d* = −2.87).

**TABLE 2:** Partial water vapor pressure gradient between the skin and air (*P*_sk,sat_–*P*_a_), rate of metabolic heat production (H_prod_), maximum evaporative capacity of the environment (*E*_max_), rate of evaporation required for heat balance (*E*_req_), rate of convective and radiative heat exchange (C + *R*), rate of respiratory convective and evaporative heat loss (C_res_ + *E*_res_), and sweating evaporative efficiency (*S*_eff_) during a 700‐kJ time trial in 33°C with Low, Moderate, high, and Very high absolute humidity. All values are expressed as means and SD. *Significantly different from Low (*p* ≤ 0.041). ^^^Significantly different from Moderate (*p* ≤ 0.035). ^+^Significantly different from High (*p* < 0.001).

Variable	Low	Moderate	High	Very high
*P*_sk,sat_–*P*_a_ gradient (kPa)	3.53 ± 0.30	2.74 ± 0.24^*^	1.99 ± 0.20^*^^	1.19 ± 0.16^*^+^
*H*_prod_ (W m^−2^)	518 ± 62	519 ± 60	497 ± 61^*^^	454 ± 63^*^+^
*E*_max_ (W m^−2^)	309 ± 26	240 ± 21^*^	175 ± 18^*^^	104 ± 14^*^+^
*E*_req_ (W m^−2^)	456 ± 58	463 ± 59	440 ± 55^^^	402 ± 54^*^+^
C + *R* (W m^−2^)	14 ± 17	16 ± 14	27 ± 12^*^^	32 ± 9^*^^
C_res_ + *E*_res_ (W m^−2^)	49 ± 7	40 ± 5^*^	30 ± 5^*^^	20 ± 3^*^+^
*S*_eff_ (ND)	0.50 ± 0.13	0.39 ± 0.10^*^	0.28 ± 0.09^*^^	0.16 ± 0.04^*^+^

*H*_prod_ was lower in Very high than Low (*p* < 0.001; *d* = −1.03), Moderate (*p* < 0.001; *d* = −1.05) and High humidity (*p* < 0.001; *d* = −0.69), and lower in High than Low (*p* = 0.041; *d* = −0.35) and Moderate humidity (*p* = 0.035; *d* = −0.36).

As humidity increased, *E*_max_ decreased and was lower in Very high than in Low (*p* < 0.001; *d* = −9.74), Moderate (*p* < 0.001; *d* = −7.63) and High humidity (*p* < 0.001; *d* = −4.43), lower in High than in Low (*p* < 0.001; *d* = −5.99) and Moderate humidity (*p* < 0.001; *d* = −3.37), and lower in Moderate than in Low humidity (*p* < 0.001; *d* = −2.88). A lower *E*_req_ was identified in Very high than in Low (*p* < 0.001; *d* = −0.96), Moderate (*p* < 0.001; *d* = −1.07) and High humidity (*p* < 0.001; *d* = −0.69), and in High than in Moderate humidity (*p* = 0.010; *d* = −0.40).

*S*_eff_ was higher in Low than Moderate (*p* = 0.003, *d* = 0.89), High (*p* < 0.001, *d* = 1.92) and Very High (*p* < 0.001, *d* = 3.39), higher in Moderate than High (*p =* 0.001, *d* = 1.20) and Very High (*p* < 0.001, *d* = 3.01), and higher in High than Very High (*p* < 0.001, *d* = 1.69).

Heat loss via *C* + *R* was greater in Very high than Low (*p* < 0.001; *d* = 1.39) and Moderate humidity (*p* < 0.001; *d* = 1.44), and in High than Low (*p* = 0.001; *d* = 0.89) and Moderate humidity (*p =* 0.008; *d* = 0.86). Heat loss via *C*_res_ + *E*_res_ was lower in Very high than Low (*p* < 0.001; *d* = −5.53), Moderate (*p* < 0.001; *d* = −5.05) and High humidity (*p* = 0.001; *d* = −2.55), lower in High than Low (*p* < 0.001; *d* = −3.16) and Moderate (*p* < 0.001; *d* = −2.08), and lower in Moderate than Low humidity (*p* < 0.001; *d* = −1.47).

### 3.4 Thermal and Cardiovascular Responses

**FIGURE 2:**
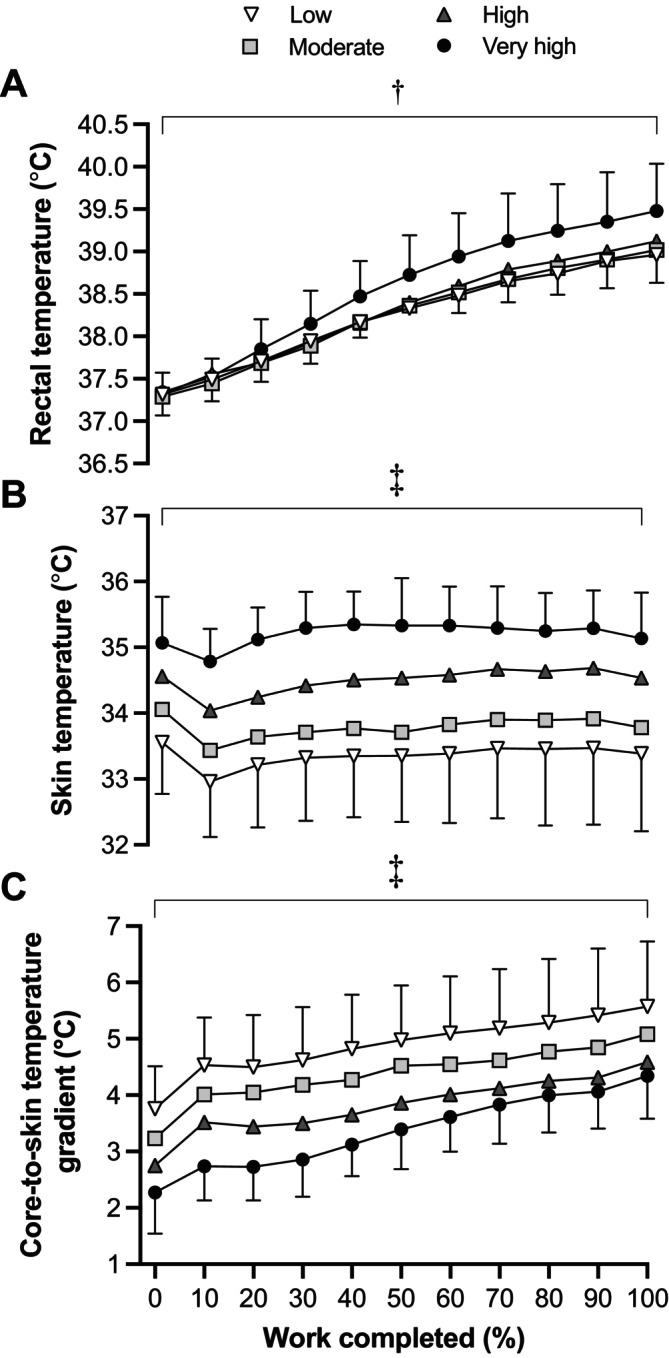
Rectal temperature (A), skin temperature (B) and core‐to‐skin temperature gradient (C) during a 700‐kJ time trial in 33°C with Low, Moderate, High, and Very high absolute humidity. ^†^Significant difference between Low and Moderate, High and Very high (*p* < 0.001). ^‡^Significant difference between all conditions (*p* < 0.001). All values are expressed as means and SD. Differences were observed using a linear mixed model.

Before entering the chamber, resting *T*_re_ was similar between conditions (mean of experimental trials: 36.94°C ± 0.34°C; *p =* 0.084). Condition (*p* < 0.001) and time (*p* < 0.001) effects were identified for mean *T*_re_, but there was no interaction effect (*p* = 0.068; Figure 2A). Mean *T*_re_ was higher in Very high (38.56°C ± 0.84°C) than in Low (38.24°C ± 0.64°C; *p* < 0.001; *d* = 0.43), Moderate (38.27°C ± 0.63°C; *p* < 0.001; *d* = 0.42) and High humidity (38.31°C ± 0.70°C; *p* < 0.001; *d* = 0.32). There was a condition effect for peak *T*_re_ (*p* < 0.001), which was higher at Very high (39.48°C ± 0.56°C) than at Low (38.97°C ± 0.44°C; *p* < 0.001; *d* = 1.01), Moderate (39.04°C ± 0.39°C; *p* = 0.002; *d* = 0.95) and High humidity (39.12°C ± 0.47°C; *p* = 0.010; *d* = 0.69). *T*_sk_ before entering the chamber was similar between conditions (mean of experimental trials: 31.80°C ± 1.15°C; *p* = 0.834). Significant condition (*p* < 0.001) and time (*p* < 0.001) effects were identified for *T*_sk_, but there was no interaction effect (*p* = 0.999; Figure 2B). *T*_sk_ was higher in Very high (35.20°C ± 0.60°C) than in Low (33.36°C ± 0.99°C; *p* < 0.001; *d* = 2.26), Moderate (33.79°C ± 0.87°C; *p* < 0.001; *d* = 1.90) and High humidity (34.49°C ± 0.72°C; *p* < 0.001; *d* = 1.08). Mean *T*_sk_ was also greater in High than in Low (*p* < 0.001; *d* = 0.88) and Moderate humidity (*p* < 0.001; *d* = 0.88), and in Moderate than in Low humidity (*p* < 0.001; *d* = 0.46). There were condition (*p* < 0.001) and time effects (*p* < 0.001), but no interaction effect (*p* = 0.999) for the *T*_re_‐to‐*T*_sk_ gradient (Figure 2C). A narrower *T*_re_‐to‐*T*_sk_ gradient was identified in Very high (3.36°C ± 0.90°C) relative to Low (4.89°C ± 1.08°C; *p* < 0.001; *d* = −1.53), Moderate (4.46°C ± 0.92°C; *p* < 0.001; *d* = −1.08) and High humidity (3.82°C ± 0.90°C; *p <* 0.001; *d* = −0.51).

**FIGURE 3:**
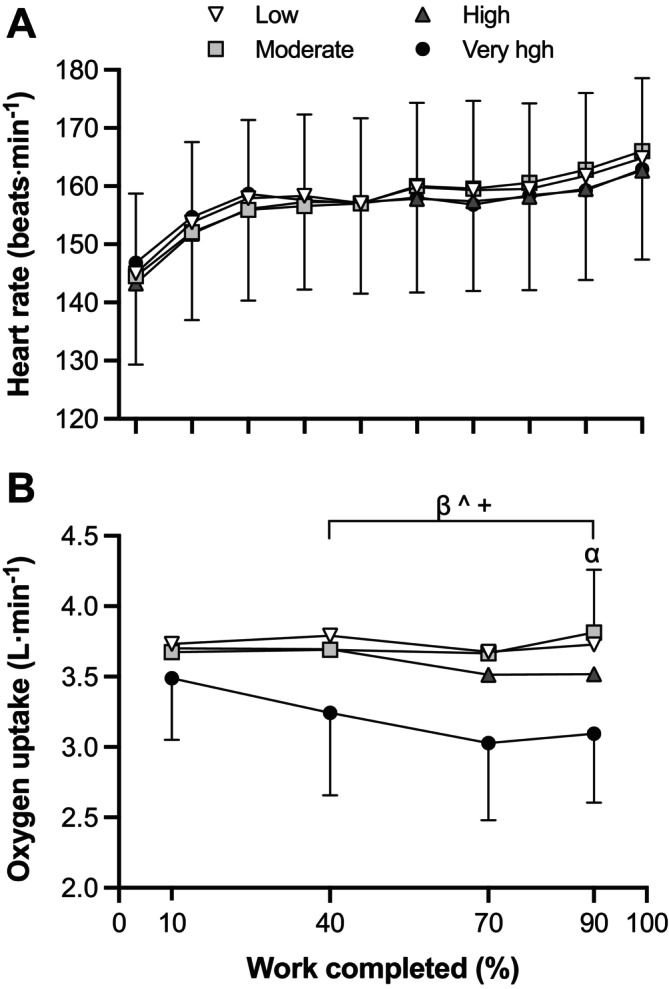
Heart rate (A) and oxygen uptake (B) during a 700‐kJ time trial in 33°C with Low, Moderate, high, and Very high absolute humidity. ^β^Significant difference between Low and Very high (*p* < 0.001). ^^^Significant difference between Moderate and Very high (*p* < 0.001). ^+^Significant difference between High and Very high (*p* < 0.001). ^α^Significant difference between Moderate and High (*p* = 0.011). All values are expressed as means and SD. Differences were observed using a linear mixed model.

Condition (*p* = 0.046) and time (*p* < 0.001) effects were identified for heart rate, but there was no interaction effect (*p* = 0.919; Figure 3A). Although there was a condition effect, no difference in mean heart rate was identified between Very high (157 ± 15 beats min^−1^), High (156 ± 16 beats min^−1^), Moderate (158 ± 15 beats min^−1^) and Low humidity (158 ± 15 beats min^−1^; all *p ≥* 0.056; *d* = −0.11 to 0.06). Condition (*p* < 0.001), time (*p* = 0.001) and interaction (*p* = 0.006) effects were identified for V̇O_2_ (Figure 3D). Mean V̇O_2_ was lower in Very high (3.2 ± 0.06 L min^−1^) than in Low (3.7 ± 0.05 L min^−1^; *p* < 0.001; *d* = −1.06), Moderate (3.7 ± 0.02 L min^−1^; *p* < 0.001; *d* = −1.01) and High humidity (3.6 ± 0.08 L min^−1^; *p* < 0.001; *d* = −0.80), and lower in High than in Low humidity (*p* = 0.025; *d* = −0.28). From 40% of work completed, V̇O_2_ was lower in Very high than in Low (*p* < 0.001; *d* = −1.36 to −1.06), Moderate (*p* < 0.001; *d* = −1.54 to −0.88) and High humidity (*p* < 0.001; *d <* = − 0.97 to −0.80). Only at 90% of work completed was V̇O_2_ lower in High than in Moderate humidity (*p* = 0.011; *d* = −0.58).

### 3.5 Perceptual and Hydration Measures

**FIGURE 4:**
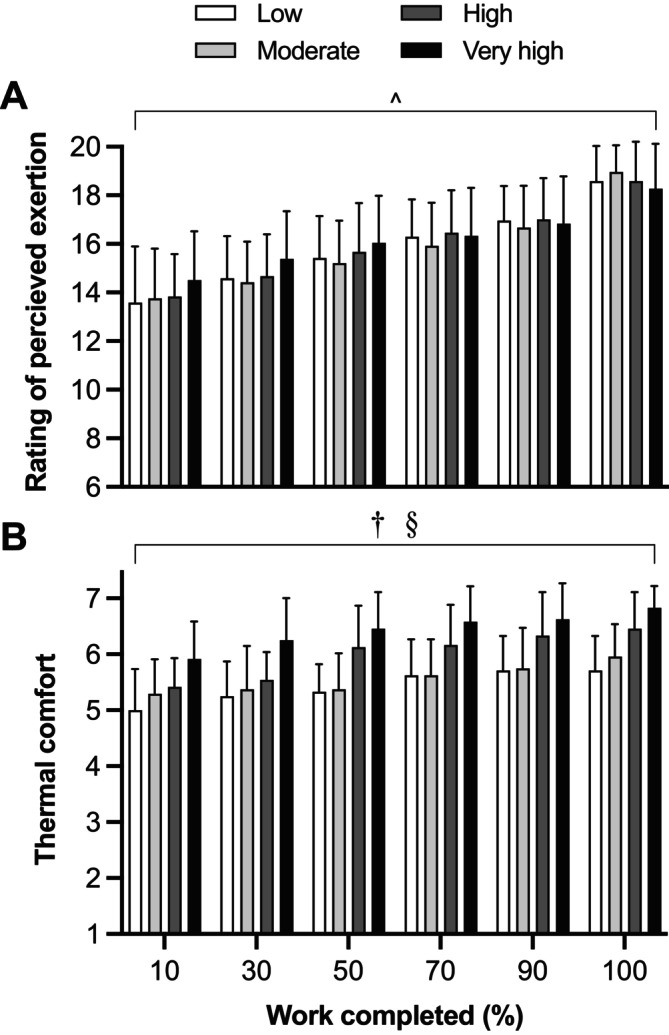
Perceptual measures during a 700‐kJ time trial in 33°C with Low, Moderate, High, and Very high absolute humidity. ^^^Significant difference between Moderate and Very high (*p* = 0.032). ^†^Significant difference between Low, Moderate, High, and Very high (*p* < 0.001). ^§^Significant difference between Low, Moderate, and High (*p* < 0.001). All values are expressed as means and SD. Differences were observed using a linear mixed model.

Condition (*p* = 0.031) and time (*p* < 0.001) effects were identified for RPE, but there was no interaction effect (*p* = 0.224; Figure 4A). RPE was only slightly greater in Very high (16.2 ± 1.9) than in Moderate (15.8 ± 1.7) humidity (*p* = 0.032; *d* = 0.16). Condition (*p* < 0.001) and time (*p* < 0.001) effects were identified for thermal comfort, but there was no interaction effect (*p* = 0.628; Figure 4B). Thermal discomfort was greater in Very high than in Low (*p* < 0.001; *d* = 1.50), Moderate (*p* < 0.001; *d* = 1.29) and High humidity (*p* < 0.001; *d* = 0.61), and in High than in Low (*p* < 0.001; *d* = 0.81) and Moderate humidity (*p* < 0.001; *d* = 0.62). Pre‐exercise body mass (mean of experimental trials: 76.50 ± 9.06 kg; *p* = 0.746) and USG (mean of experimental trials: 1.01 ± 0.01; *p* = 0.914) were similar across all conditions. A significant condition effect was identified for fluid consumption (*p* = 0.007), with more fluid consumed in Very high (1.08 ± 0.34 L) than in Low (0.78 ± 0.34 L, *p* = 0.010; *d* = 0.88) and Moderate humidity (0.83 ± 0.41 L, *p* = 0.028; *d* = 0.71), but not in High (0.92 ± 0.29 L, *p* = 0.823; *d* = 0.43). There was no condition effect for whole‐body sweat rate (*p* = 0.636) which was 1.91 ± 0.42, 1.90 ± 0.54, 1.98 ± 0.54, and 2.03 ± 0.51 L h^−1^ in Low, Moderate, High, and Very high humidity, respectively. There was also no condition effect for the percentage of body mass loss (*p* = 0.958), which was 0.89% ± 0.33%, 0.82% ± 0.30%, 0.84% ± 0.47%, and 0.88% ± 0.59% in Low, Moderate, High, and Very high humidity, respectively.

## 4 Discussion

This study investigated the effects of Low (1.6 kPa), Moderate (2.5 kPa), High (3.5 kPa), and Very high (4.5 kPa) humidity levels on self‐paced exercise performance in 33°C, along with the associated changes in heat exchange, thermal, cardiovascular, and perceptual strain. Our data demonstrate that performance was similar in the drier conditions (Low: 260 W; Moderate: 257 W), but impaired by ~5% in High humidity (246 W) and further compromised in Very high humidity (~16%, 222 W; Figure 1). Power output was also lower from the onset of the time trial in the Very high compared to Low humidity condition, highlighting the immediate effect of elevated humidity on work rate and pacing in hot conditions. The lower mean power output maintained in High and Very high humidity was associated with a narrower *P*_sk,sat_–*P*_a_ gradient and lower *E*_max_ and *S*_eff_ (Table 2), resulting in greater thermal strain despite the lower *H*_prod_. Correspondingly, a similar heart rate was observed across all conditions (Figure 4), suggestive of the maintenance of a similar relative exercise intensity. These data indicate that elevated humidity (> 2.5 kPa) impairs self‐paced exercise performance in the heat due to reductions in evaporative potential and efficiency, and the consequent increase in physiological and perceptual strain.

### 4.1 Time Trial Performance

In the current study, mean power output was similar in the drier conditions of Low and Moderate humidity, with power output maintained within ~7 W between conditions throughout the time trials. These findings support previous constant work rate exercise studies examining systematic increases in humidity during exercise in 30°C–31°C and demonstrating a similar time to exhaustion between absolute humidities of 1.02 and 2.34 kPa [6, 7]. Our findings also indicate that an increase in absolute humidity beyond 2.5 kPa (i.e., Moderate) leads to a self‐paced exercise performance decrement. This observation extends that of Jenkins, Campbell, Lee, Mundel, and Cotter [8] showing a decrease in time trial performance in 36°C when absolute humidity increased from 1.96 to 3.96 kPa [8]. In the current study, power output was lower in the High than Low humidity condition at 60% of work completed, and from 90% of work completed, it was lower in High than Low and Moderate. Of note, power output was lower from the onset of exercise (10% of work completed) in the Very high humidity condition compared with Low. This initially lower work rate continued to decrease until the final end‐spurt (Figure 1). In contrast, in our recent comparison of the impact of dry‐bulb temperature (13°C, 20°C, 28°C and 36°C) on 30 km cycling time trial performance, power output was similar across conditions for the first 3 km, decreasing from 9 km in 36°C relative to 13°C and 20°C, and decreasing in the 36°C condition relative to 28°C from 12 km onwards [10]. These data indicate that the effect of elevated humidity on self‐paced exercise performance in hot conditions may be more immediate than that of temperature. The lower initial work rate in the Very high humidity condition may represent an attempt to maintain an optimal performance intensity [9, 11], similar to that of the drier environments, as demonstrated by the similar heart rate and RPE between conditions (Figure 4A). It may also be associated with higher *T*_sk_ and thermal discomfort (Figures 2B and 4B) and an attempt to improve thermal perception and perceived exertion, or an experience‐based decision to adopt a more conservative pacing strategy to avoid large eventual decrements in work rate [21, 22].

Overall, our data indicate that power output is similarly maintained in hot (33°C) environments with Low and Moderate humidity, but that reductions in performance occur when absolute humidity exceeds 2.5 kPa, in association with a lower *E*_max_ and subsequent increase in thermal and cardiovascular strain. For context, an absolute humidity of 2.5 kPa is associated with different combinations of ambient temperature and RH (e.g., 27°C and 70% RH, 30°C and 59% RH, 33°C and 50% RH), which can occur in different geographical locations (e.g., Atlanta, USA; Brisbane, Australia; Tokyo, Japan). Of note, air flow in the current study was ~3.4 m s^−1^ (~12 km h^−1^), which is appreciably less than what trained cyclists experience during an outdoor time trial. It is well established that air flow improves heat dissipation via convection and evaporation in conditions where *T*_sk_ is higher than ambient temperature [3, 23], which reduces thermal strain and improves endurance capacity [24, 25]. However, we have recently shown that air flows of 30 and 44 km h^−1^ during a 700‐kJ time trial in 32°C and 40% RH did not improve heat dissipation compared to an air flow of 16 km h^−1^ because of the attainment of maximum *S*_eff_ [26]. Consequently, performance was also not improved at the higher air flows relative to the 16 km h^−1^ condition when compared with a still air condition. Similar findings were reported by Saunders, Dugas, Tucker, Lambert, and Noakes [25], who noted that thermoregulatory capacity, rather than the environment, limited thermal balance as environmental heat dissipating capacity increased with higher air flows (0 to 50 km h^−1^). As such, it is unlikely that increasing air flow in the current study would have substantially impacted the development of thermal strain and subsequently performance. Nevertheless, understanding the impact of absolute humidity on health and performance and identifying competition locations where it may be a factor are important considerations for athletes aiming to optimize performance.

### 4.2 Heat Balance and Thermal Strain

Humid environments limit the potential for evaporative heat loss and increase thermal strain during work and exercise [5, 7, 27]. The systematic reduction in the *P*_sk,sat_–*P*_a_ gradient with increases in humidity in the current study was matched by a decrement in *E*_max_. The reduction in *E*_max_ coincided with a slightly greater dry heat loss (i.e., *C* + *R*) due to a higher *T*_sk_ widening the skin‐to‐air temperature gradient in the High and Very high humidity conditions (Table 2). However, since the evaporation of sweat is the main avenue for heat loss under heat stress, the greater dry heat loss at higher humidities did not compensate for the reduction in *E*_max_, and thus did not attenuate the rise in thermal strain. Accordingly, the progressive narrowing of the *P*_sk,sat_–*P*_a_ gradient with higher humidity appears to have led to an inability to evaporate sweat from the skin surface. Indeed, our data suggest that the development of thermal strain during self‐paced exercise in the heat with increasingly elevated humidity is related to a lowering of *S*_eff_ (i.e., the proportion of sweat production that contributes to evaporative cooling) due to a growing layer of sweat coalescing and dripping off the skin, rather than an inability to produce sweat [5, 28, 29]. This suggestion is supported by similar whole‐body sweat rates between conditions (1.91 to 2.03 L h^−1^) and decreasing *S*_eff_ from Low to Very High humidity (Table 2).

In the Low, Moderate, and High humidity conditions, peak *T*_re_ was similar despite mean power output being ~11 W lower in High, which corresponded to an ~22 W lower *H*_prod_ compared to the drier conditions (Table 2). An increase in humidity from High to Very high led to an even larger performance decrement (~22 W) and *H*_prod_ (~43 W). However, the lower *H*_prod_ did not compensate for the detrimental impact of humidity on evaporative capacity in the Very high humidity condition, which is reflected in the higher peak *T*_re_ (by ~0.43°C) compared with all other humidity conditions. In contrast, when completing a time to exhaustion test at 70% V̇O_2peak_ in 24%, 40%, 60%, and 80% RH, *T*_re_ at exhaustion was similar across all conditions, although the rate of increase was greater with elevated humidity [6]. Our findings support those reported for constant work rate exercise in that humid environments, especially > 2.5 kPa, exacerbate thermal strain and impair exercise performance.

### 4.3 Thermal and Cardiovascular Strain

Impairments in exercise performance in the heat are associated with an increased cardiovascular response subsequent to elevations in thermal strain [30]. The progressive elevation in thermal and cardiovascular strain gradually reduces V̇O_2peak_ and increases the relative exercise intensity (i.e., %V̇O_2peak_) for work performed at a given absolute work rate [9, 11]. In the current study, elevated thermal strain was evidenced by a higher *T*_re_ and a narrow *T*_re_‐to‐*T*_sk_ gradient (Figure 2), whereas cardiovascular strain was reflected in a similar heart rate response (~90% of maximum heart rate) across conditions (Figure 3). A comparable heart rate response across all conditions suggests the maintenance of a similar relative exercise intensity, but for a lower absolute work rate (i.e., power output) at higher humidity levels. The similar RPE between conditions further supports the maintenance of a similar relative exercise intensity across humidity conditions (Figure 4A). The exacerbated cardiovascular response under heat stress has been shown to progressively reduce V̇O_2peak_ [31, 32] and decrease work rate during self‐paced exercise in order to maintain an optimal performance intensity (i.e., % V̇O_2peak_ [11, 12]). Our data support this premise and demonstrate that reductions in evaporative potential during self‐paced cycling in hot and humid conditions exacerbate thermal strain (i.e., narrow *T*_re_‐to‐*T*_sk_ gradient) and concomitantly the cardiovascular response, resulting in a marked impairment of performance (i.e., mean power output).

## 5 Conclusion

This study examined the impact of different absolute humidity levels on prolonged self‐paced exercise in 33°C. Performance was similar at the lower humidity levels (i.e., Low and Moderate), but impaired in High and further compromised in Very high humidity. In the higher humidity environments, decrements in work rate relative to the lower humidity environments occurred from the early stages of exercise. In Very high humidity, work rate was lower from the commencement of exercise compared to Low humidity, possibly to maintain an optimal performance intensity, improve thermal perception and perceived exertion, or to avoid large eventual decrements in work rate by adopting a conservative pacing strategy. Notwithstanding, the impairment in performance in High and Very high humidity conditions was associated with a greater thermal strain, manifested as a narrower *T*_re_‐to‐*T*_sk_ temperature gradient. This led to the maintenance of a similar relative exercise intensity across all humidity conditions, as indicated by the similar heart rate responses, but for a lower absolute work rate (i.e., power output) in the High and Very high humidity conditions. The greater thermal strain in the higher humidity conditions was the result of a lower capacity for evaporative heat loss, due to the narrow *P*_sk,sat_–*P*_a_ gradient and reduced *S*_eff_.

## 6 Perspective

Our study demonstrates the impact of humidity on heat dissipation during self‐paced exercise in the heat and the subsequent exacerbation of the physiological and perceptual cascade that leads to an inability to maintain an elevated work rate. These data, along with our previous work examining the role of ambient temperature [10] and air flow [26], as well as research examining the impact of radiant heat [33], highlight the need to consider all environmental parameters when preparing athletes to compete in challenging conditions. As athletes continue to compete in the heat at high intensities, a greater understanding of how environmental heat stress, driven by different parameters, impacts heat balance and performance will enable the development of more targeted preparation strategies (e.g., hydration, cooling, heat acclimation) aimed at optimizing performance. Importantly, these strategies must balance performance optimization and disruption. For example, fluid consumption in the Very high humidity condition (1.08 L) of the current study was significantly greater than in the Low (0.78 L) and Moderate (0.83 L) conditions, which allowed for a similar percentage of body mass loss (~0.86%). However, greater fluid consumption during an outdoor time trial would lead to breaking aerodynamic position more often to drink and compromise performance. As such, careful consideration must be placed on the implementation of specific strategies, as well as evaluation of their effectiveness.

## Author Contributions

F.M.B., B.C., O.J., and J.D.P. conceived and designed research; F.M.B. performed experiments; F.M.B., B.C., and J.D.P. interpreted results of experiments; F.M.B. prepared figures; F.M.B. drafted manuscript; F.M.B., B.C., O.J., and J.D.P. edited and revised manuscript; and F.M.B., B.C., O.J., and J.D.P. approved final version of manuscript.

## Conflicts of Interest

The authors declare no conflicts of interest.

## Data Availability

Data are available from the corresponding author (julien.periard@canberra.edu.au) upon reasonable request and a signed access agreement.

## Appendix A

Calculation 1. Maximum evaporative capacity of the environment (*E*_max_) [34, 35]:

$$E_{\max}=0.84\;\cdot \;\frac{\left(P_{\mathrm{sk},\mathrm{sat}}-P_{a}\right)}{\left(R_{e,\mathrm{cl}}+\frac{1}{h_{e}∙f_{\mathrm{cl}}}\right)}\;\left(W\;m^{-2}\right)$$

where 0.84 is the maximum skin wettedness of a trained but non‐heat‐acclimated individual [36], *P*_sk,sat_ is the saturated water vapor pressure at skin temperature (kPa) and can be derived using Antoine's equation [37]:

$$P_{\mathrm{sk},\mathrm{sat}}=\frac{\mathrm{EXP}\left[18.956-\frac{4030.18}{T_{\mathrm{sk}}+235}\;\right]}{10}\;\left(\mathrm{kPa}\right)$$

*P*_a_ is the ambient water vapor pressure (kPa), *R*_e,cl_ is the evaporative heat transfer resistance of clothing and is assumed to be 0.0075 m^2^ kPa W^−1^, *h*_e_ is the evaporative heat transfer coefficient and was estimated as:

$$h_{e}=16.5\;\cdot \;h_{c}\;\left(W\;m^{-2}\;{\mathrm{kPa}}^{-1}\right)$$

where 16.5 is the Lewis Relation coefficient, *h*_c_ is the convective heat transfer coefficient, and it was calculated as [38]:

$$h_{c}=8.3\;{\left(0.07+0.0043\cdot f_{\mathrm{ped}}+v_{\mathrm{air}}\right)}^{0.86}\cdot {\left(\frac{P_{d}}{760}\;\right)}^{0.55}\;\left(W\;m^{-2}\;K^{-1}\right)$$

where *f*_ped_ is pedaling frequency (revolutions/min) and *v*_*air*_ is air velocity (m s^−1^). Note: As testing was conducted above sea level, *h*_*c*_ was corrected for the reduction in barometric pressure.

*f*_cl_ is the clothing area factor defined as the surface area of the clothed body divided by the surface area of the nude body and is estimated using [39]:

$$f_{\mathrm{cl}}=1+\left(0.31\cdot I_{\mathrm{cl}}\right)\;\left(\mathrm{ND}\right)$$

where *I*_*cl*_ is intrinsic clothing insulation for the clothing ensemble, which was set at 0.2.

Calculation 2. Rate of evaporation required for heat balance (*E*_req_):

$$E_{\mathrm{req}}=H_{\text{prod}}–\left(C_{\mathrm{sk}}+R_{\mathrm{sk}}\right)–\left(C_{\mathrm{res}}+E_{\mathrm{res}}\right)\;\left(W\;m^{-2}\right)$$

where *H*_prod_ is the rate of metabolic heat production and was calculated as:

$$H_{\text{prod}}=\frac{M-W}{\begin{array}{c} \mathrm{AD} \end{array}}$$

where *W* is the rate of mechanical work (i.e., power output; W) and *M* is the metabolic rate, which was estimated by measuring the rate of oxygen consumption and carbon dioxide production using the equation:

$$M=VO_{2}\;\frac{\left[\left(\left(\frac{\mathrm{RER}-0.7}{0.3}\right)\cdot e_{c}\right)+\left(\left(\frac{1.0-\mathrm{RER}}{0.3}\right)\cdot e_{f}\right)\right]}{60}1000\;\left(W\right)$$

VO_2_ is the rate of oxygen consumption (L min^−1^). RER is the ratio of carbon dioxide production to oxygen consumption. *e*_c_ is the caloric equivalent per liter of oxygen for carbohydrate oxidation (21.13 kJ) and *e*_f_ is the caloric equivalent per liter of oxygen for fat oxidation (19.62 kJ). To normalize *M* in W m^−2^ it was divided by body surface area (*A*_D_) using the Dubois and Dubois equation [40]:

$$A_{D}=0.202\;\cdot \;{\text{mass}}^{0.425}\;\cdot \;{\text{height}}^{0.725}\;\left(m^{2}\right)$$

Mass (kg) and height (m).

Where (*C*_sk_ *+ R*_sk_) is dry heat exchange from the skin and is calculated as the sum of convective (*C*) and radiative (*R*) heat transfer from the skin using the equation:

$$C_{\mathrm{sk}}+R_{\mathrm{sk}}=\frac{\left(T_{\mathrm{sk}}-T_{o}\right)}{\left(R_{\mathrm{cl}}+\frac{1}{h∙f_{\mathrm{cl}}}\right)}\;\;\left(W\;m^{-2}\right)$$

*T*_sk_ is skin temperature and *T*_o_ is operative temperature:

*R*_cl_ is the dry heat transfer resistance of clothing and was calculated as:

$$R_{\mathrm{cl}}=0.155\;\cdot \;I_{\mathrm{cl}}\;\left(m^{2{}^{\circ}}C\;W^{-1}\right)$$

*h* is the combined heat transfer coefficient (*h*_c_ + *h*_r_) where *h*_r_ is the radiative heat transfer coefficient estimated using:

$$h_{r}=4\mathrm{ɛ\sigma}\;\frac{A_{r}}{A_{D}}\;{\left[273.2+\frac{T_{sk}\;+T_{r}}{2}\;\right]}^{3}\;\left(W\;m^{-2}\;K^{-1}\right)$$

*ɛ* is the emissivity of the body surface (0.95). *σ* is the Stefan‐Boltzmann constant (5.67 × 10^−8^ W m^−2^ K^−1^). *A*_r_ */A*_D_ is the effective radiative area of the body (0.7 m^2^) for a standing person. *T*_r_ is the mean radiant temperature (°C) which was equivalent to the dry‐bulb temperature as tests were conducted indoors.

*C*_res_ *+ E*_res_ is respiratory heat exchange and was calculated as the sum of convective (*C*) and radiative (*R*) heat transfer from respiration using the equation:

$$\begin{array}{c} C_{\mathrm{res}}+E_{\mathrm{res}}=\left(0.001516\;\cdot \;M\;\cdot \;\left(28.56+0.641\;\cdot \;P_{a}-0.885\;\cdot \;T_{a}\right)\right)\\ \;+\left(0.00127\;\cdot \;M\cdot \;\left(59.34+0.53\;\cdot \;T_{a}-11.63\;\cdot \;P_{a}\right)\right)\;\left(W\;m^{-2}\right) \end{array}$$

Calculation 3. Sweating evaporative efficiency (*S*_eff_):

$$S_{\mathrm{eff}}=\frac{E_{\max}\;\cdot \;A_{D}}{1000\;\cdot \;\frac{\text{WBSR}\;\cdot \;2426\;}{3600}\;}\;\;\left(\mathrm{ND}\right)$$

where WBSR is the whole‐body sweat rate (L h^−1^) and 2426 is the latent heat of vaporization (J g^−1^).

## References

[1] M. Nielsen , “Die Regulation der Körpertemperatur bei Muskelabeit,” Skandinavisches Archiv Für Physiologie 79, no. 2 (1938): 193–230.

[2] A. P. Gagge , “A New Physiological Variable Associated With Sensible and Insensible Perspiration,” American Journal of Physiology‐Legacy Content 1220 (1937): 277–287.

[3] A. P. Gagge and R. R. Gonzalez , “Mechanisms of Heat Exchange: Biophysics and Physiology,” in Handbook of Physiology: Environmental Physiology (American Physiological Society, 1996), 45–84.

[4] R. R. Gonzalez , K. B. Pandolf , and A. P. Gagge , “Heat Acclimation and Decline in Sweating During Humidity Transients,” Journal of Applied Physiology 36, no. 4 (1974): 419–425. 4820323 10.1152/jappl.1974.36.4.419

[5] B. Alber‐Wallerström and I. Holmér , “Efficiency of Sweat Evaporation in Unacclimatized Man Working in a Hot Humid Environment,” European Journal of Applied Physiology and Occupational Physiology 54, no. 5 (1985): 480–487, 10.1007/bf00422956. 4085475

[6] R. J. Maughan , H. Otani , and P. Watson , “Influence of Relative Humidity on Prolonged Exercise Capacity in a Warm Environment,” European Journal of Applied Physiology 112, no. 6 (2012): 2313–2321, 10.1007/s00421-011-2206-7. 22012542

[7] A. M. Che Muhamed , K. Atkins , S. R. Stannard , T. Mundel , and M. W. Thompson , “The Effects of a Systematic Increase in Relative Humidity on Thermoregulatory and Circulatory Responses During Prolonged Running Exercise in the Heat,” Temperature 3, no. 3 (2016): 455–464, 10.1080/23328940.2016.1182669. PMC5079215 28349085

[8] E. J. Jenkins , H. A. Campbell , J. K. W. Lee , T. Mundel , and J. D. Cotter , “Delineating the Impacts of Air Temperature and Humidity for Endurance Exercise,” Experimental Physiology 108, no. 2 (2023): 207–220, 10.1113/EP090969. 36537856 PMC10103870

[9] J. D. Périard , M. N. Cramer , P. G. Chapman , C. Caillaud , and M. W. Thompson , “Cardiovascular Strain Impairs Prolonged Self‐Paced Exercise in the Heat,” Experimental Physiology 96, no. 2 (2011): 134–144, 10.1113/expphysiol.2010.054213. 20851861

[10] F. M. Bright , B. Clark , O. Jay , and J. D. Périard , “The Effect of Minimal Differences in the Skin‐to‐Air Vapor Pressure Gradient at Various Dry‐Bulb Temperatures on Self‐Paced Exercise Performance,” Journal of Applied Physiology 131, no. 3 (2021): 1176–1185, 10.1152/japplphysiol.01059.2020. 34323591

[11] J. D. Périard and S. Racinais , “Self‐Paced Exercise in Hot and Cool Conditions Is Associated With the Maintenance of %VO_2peak_ Within a Narrow Range,” Journal of Applied Physiology 2015, no. 118 (2015): 1258–1265, 10.1152/japplphysiol.00084.2015. 25814635

[12] J. D. Périard and S. Racinais , “Performance and Pacing During Cycle Exercise in Hyperthermic and Hypoxic Conditions,” Medicine and Science in Sports and Exercise 48, no. 5 (2016): 845–853, 10.1249/MSS.0000000000000839. 26656777

[13] H. Beal , J. Corbett , D. Davis , and M. J. Barwood , “Marathon Performance and Pacing in the Doha 2019 Women's IAAF World Championships: Extreme Heat, Suboptimal Pacing, and High Failure Rates,” International Journal of Sports Physiology and Performance 17, no. 7 (2022): 1119–1125, 10.1123/ijspp.2022-0020. 35580843

[14] WeatherUnderground , “Tokyo Olympics Men's Cycling Road Race Data From Musashino (11:00), Atsugi (14:00) and Fuji Speedway (17:00),” (2020), https://www.wunderground.com.

[15] K. De Pauw , B. Roelands , S. S. Cheung , B. de Geus , G. Rietjens , and R. Meeusen , “Guidelines to Classify Subject Groups in Sport‐Science Research,” International Journal of Sports Physiology and Performance 2013, no. 8 (2013): 111–122. 10.1123/ijspp.8.2.111 23428482

[16] ESSA , “Exercise & Sports Science Australia Adult Pre‐Exercise Screening System (APSS),” Exercise & Sports Science Australia.

[17] N. L. Ramanathan , “A New Weighting System for Mean Surface Temperature of the Human Body,” Journal of Applied Physiology 19 (1964): 531–533. 14173555 10.1152/jappl.1964.19.3.531

[18] G. A. Borg , “Psychophysical Bases of Perceived Exertion,” Medicine and Science in Sports and Exercise 14, no. 5 (1982): 377–381. 7154893

[19] T. Bedford , “The Warmth Factor in Comfort at Work: A Physiological Study of Heating and Ventilation,” (1936): 35.

[20] J. Cohen , “A Power Primer,” Psychological Bulletin 112, no. 1 (1992): 155–159. 19565683 10.1037//0033-2909.112.1.155

[21] A. D. Flouris and Z. J. Schlader , “Human Behavioral Thermoregulation During Exercise in the Heat,” Scandinavian Journal of Medicine & Science in Sports 2015, no. 25 (2015): 52–64, 10.1111/sms.12349. 25943656

[22] Z. J. Schlader , S. E. Simmons , S. R. Stannard , and T. Mundel , “The Independent Roles of Temperature and Thermal Perception in the Control of Human Thermoregulatory Behavior,” Physiology & Behavior 103, no. 2 (2011): 217–224, 10.1016/j.physbeh.2011.02.002. 21315099

[23] W. C. Adams , G. W. Mack , G. W. Langhans , and E. R. Nadel , “Effects of Varied Air Velocity on Sweating and Evaporative Cooling Rates During Exercise,” Journal of Applied Physiology 73, no. 6 (1992): 2668–2674. 1490985 10.1152/jappl.1992.73.6.2668

[24] H. Otani , M. Kaya , A. Tamaki , P. Watson , and R. J. Maughan , “Air Velocity Influences Thermoregulation and Endurance Exercise Capacity in the Heat,” Applied Physiology, Nutrition, and Metabolism 43 (2017): 131–138, 10.1139/apnm-2017-0448. 28985477

[25] A. G. Saunders , J. P. Dugas , R. Tucker , M. I. Lambert , and T. D. Noakes , “The Effects of Different Air Velocities on Heat Storage and Body Temperature in Humans Cycling in a Hot, Humid Environment,” Acta Physiologica Scandinavica 183, no. 3 (2005): 241–255. 15743384 10.1111/j.1365-201X.2004.01400.x

[26] F. M. Bright , B. Clark , O. Jay , and J. D. Périard , “Influence of Air Velocity on Self‐Paced Exercise Performance in Hot Conditions,” Medicine & Science in Sports & Exercise 55, no. 8 (2023): 1382–1391, 10.1249/mss.0000000000003168. 36989528

[27] J. Sen Gupta , Y. V. Swamy , G. Pichan , and G. P. Dimri , “Physiological Responses During Continuous Work in Hot Dry and Hot Humid Environments in Indians,” International Journal of Biometeorology 28, no. 2 (1984): 137–146, 10.1007/bf02191726. 6735516

[28] V. Candas , J. P. Libert , and J. J. Vogt , “Influence of Air Velocity and Heat Acclimation on Human Skin Wettedness and Sweating Efficiency,” Journal of Applied Physiology 47, no. 6 (1979): 1194–1200, 10.1152/jappl.1979.47.6.1194. 536289

[29] G. Havenith and D. Fiala , “Thermal Indices and Thermophysiological Modeling for Heat Stress,” Comprehensive Physiology 6, no. 1 (2015): 255–302, 10.1002/cphy.c140051. 26756633

[30] J. D. Périard , T. M. H. Eijsvogels , and H. A. M. Daanen , “Exercise Under Heat Stress: Thermoregulation, Hydration, Performance Implications and Mitigation Strategies,” Physiological Reviews 101, no. 4 (2021): 1873–1979, 10.1152/physrev.00038.2020. 33829868

[31] S. A. Arngrimsson , D. S. Petitt , F. Borrani , K. A. Skinner , and K. J. Cureton , “Hyperthermia and Maximal Oxygen Uptake in Men and Women,” European Journal of Applied Physiology 92, no. 4–5 (2004): 524–532, 10.1007/s00421-004-1053-1. 15150660

[32] S. A. Arngrimsson , D. J. Stewart , F. Borrani , K. A. Skinner , and K. J. Cureton , “Relation of Heart Rate to Percent VO2 Peak During Submaximal Exercise in the Heat,” Journal of Applied Physiology 94, no. 3 (2003): 1162–1168, 10.1152/japplphysiol.00508.2002. 12391114

[33] H. Otani , M. Kaya , A. Tamaki , P. Watson , and R. J. Maughan , “Effects of Solar Radiation on Endurance Exercise Capacity in a Hot Environment,” European Journal of Applied Physiology 116, no. 4 (2016): 769–779. 26842928 10.1007/s00421-016-3335-9

[34] K. Parsons , “The Effects of Hot, Moderate and Cold Environments on Human Health, Comfort and Performance,” in Human Thermal Environments, 2nd ed. (Taylor & Francis, 2003).

[35] ASHRAE , “Thermal Comfort,” in ASHRAE Handbook of Fundamentals, ed. R. A. Parsons (American Society of Heating, Refrigerating and Air‐Conditioning Engineers, 1997).

[36] N. Ravanelli , G. B. Coombs , P. Imbeault , and O. Jay , “Maximum Skin Wettedness After Aerobic Training With and Without Heat Acclimation,” Medicine and Science in Sports and Exercise 50, no. 2 (2018): 299–307, 10.1249/mss.0000000000001439. 28991042

[37] G. W. Thomson , “The Antoine Equation for Vapor‐Pressure Data,” Chemical Reviews 38 (1946): 1–39. 21016992 10.1021/cr60119a001

[38] W. A. Lotens and G. Havenith , “Calculation of Clothing Insulation and Vapour Resistance,” Ergonomics 34, no. 2 (1991): 233–254, 10.1080/00140139108967309.

[39] E. A. J. McCullough , B. W. Jones , and J. Huck , “A Comprehensive Database for Estimating Clothing Insulation,” ASHRAE Transactions 91 (1985): 29–47.

[40] D. DuBois , “A Formula to Estimate Surface Area if Height and Weight Are Known,” Archives of Internal Medicine 17 (1916): 863–871.

